# The mesopelagic anoxic Black Sea as an unexpected habitat for *Synechococcus* challenges our understanding of global “deep red fluorescence”

**DOI:** 10.1038/s41396-019-0378-z

**Published:** 2019-02-28

**Authors:** Cristiana Callieri, Violeta Slabakova, Nina Dzhembekova, Nataliya Slabakova, Elisaveta Peneva, Pedro J. Cabello-Yeves, Andrea Di Cesare, Ester M. Eckert, Roberto Bertoni, Gianluca Corno, Michaela M. Salcher, Lyudmila Kamburska, Filippo Bertoni, Snejana Moncheva

**Affiliations:** 1 National Research Council CNR-IRSA, Microbial Ecology Group, Verbania, Italy; 2 Institute of Oceanology “Fridtjof Nansen” – Bulgarian Academy of Sciences, Varna, Bulgaria; 3 Sofia University “St. Kliment Ohridski”, Faculty of Physics, Sofia, Bulgaria; 4 Evolutionary Genomics Group, Departamento de Producción Vegetal y Microbiología, Universidad Miguel Hernández, San Juan de Alicante, Spain; 5 Department of Aquatic Microbial Ecology, Institute of Hydrobiology, Biology Certer CAS, Ceske Budejovice, Czech Republic; 6 Limnological Station, Institute of Plant and Microbial Biology, University of Zurich, Kilchberg, Switzerland; 7 Museum für Naturkunde, Berlin, Germany

**Keywords:** Phylogenomics, Microbial ecology, Molecular biology

## Abstract

The Black Sea is the largest meromictic sea with a reservoir of anoxic water extending from 100 to 1000 m depth. These deeper layers are characterised by a poorly understood fluorescence signal called “deep red fluorescence”, a chlorophyll *a*- (Chl *a*) like signal found in deep dark oceanic waters. In two cruises, we repeatedly found up to 10^3^ cells ml^−1^ of picocyanobacteria at 750 m depth in these waters and isolated two phycoerythrin-rich *Synechococcus* sp. strains (BS55D and BS56D). Tests on BS56D revealed its high adaptability, involving the accumulation of Chl *a* in anoxic/dark conditions and its capacity to photosynthesise when re-exposed to light. Whole-genome sequencing of the two strains showed the presence of genes that confirms the putative ability of our strains to survive in harsh mesopelagic environments. This discovery provides new evidence to support early speculations associating the “deep red fluorescence” signal to viable picocyanobacteria populations in the deep oxygen-depleted oceans, suggesting a reconsideration of the ecological role of a viable stock of *Synechococcus* in dark deep waters.

## Introduction

The Black Sea is known for its peculiar vertical structure: a strong stratification prevents deep-water ventilation, leading to complete absence of oxygen and abundance of hydrogen sulphide and ammonia in its deep layers [1–3]. The oxycline is quite shallow (~100 m) and coincides approximately with the halocline and winter convection depth [4, 5]. Consequently, most biological activity, light and oxygen dependent, is confined to the first 100 m from the surface, while the deeper layers constitute an unfavourable environment for photoautotrophic microorganisms [6, 7]. Since 2013 several Bio-Argo autonomous profiling floats were deployed in the Black Sea, providing real-time profiles to a depth of 1000 m for several variables (http://www.oao.obs-vlfr.fr/bioargo/PHP/basbio001d/basbio001d.htm). The floats consistently measured a chlorophyll *a* (Chl *a*) concentration increase between 100 and 1000 m depth, not visible in the corresponding profiles for the Mediterranean Sea and the Atlantic Ocean. This increase of red fluorescence well below the euphotic zone was firstly described in the early 1980s [8, 9], during oceanographic research in different areas of the Pacific Ocean: from the subtropical (off Mexico, between California and Hawai [1, 9]) to the North Pacific [10]. This scattering in the visible wavelengths at around 415 nm, termed “deep red fluorescence”, has been found associated with the oxygen minimum zone (OMZ) [9, 11] and was described as a feature of the global oceans, since it was reported across different oceanic regions [12].

Since its detection, this signal caught scientists’ attention: how to understand a consistent and widespread increase in Chl *a* in deep dark waters where photosynthesis is precluded? Answering this question through the study of fluorescent spectra, the hypothesis was formulated that “deep red fluorescence” could be attributed to the presence of a specific chromophore/fluorophore of pigment origin, possibly derived from autotrophic or heterotrophic microorganisms [12, 13]. Associating the signal with high CDOM and non-algal matter concentration, though, led some authors to treat non-zero Chl *a* values at depth as an artefact caused by CDOM presence and to propose techniques for correcting fluorometric chlorophyll measures at depth [14, 15]. This interpretation of “deep red fluorescence” is grounded in the widespread understanding of deep, dark, anoxic waters as a prohibitive environment for photoautotrophs. However, prior studies suggesting that this signal could derive from picocyanobacteria [12] led us to consider and explore the presence of *Synechococcus* in these deep waters. Picocyanobacteria of the genus *Synechococcus* are cosmopolitan unicellular microorganisms (picoplankton size range: 0.2–2 µm) abundant across a wide spectrum of trophic conditions in lakes and oceans [16, 17]. Yet, studies on the spatio-temporal dynamics of *Synechococcus* populations have been generally restricted to the photic layer, as the genus is composed of autotrophic species. Low numbers of *Synechococcus* cells have been found in subtropical NW Pacific in meso- and bathypelagic waters, transported from the epipelagic zone down the water column [18]. But, despite their well-documented structural plasticity [19] and ecological adaptability to extreme environments [20], their presence was never reported before in deep anoxic meso- and bathypelagic waters, like the deep zone of the Black Sea. Here, the simultaneous absence of oxygen and presence of ammonia and hydrogen sulphate creates particularly harsh environmental conditions for aerobic photoautotrophs.

The recovery of picocyanobacteria in the deep Black Sea at first sight appears to corroborate early speculations that related the Chl *a* increase to viable populations of PE-rich photoautotrophic cyanobacteria [1]. Following these and other suggestions that indicate a relation between the fluorescence signal and these organisms [13], we advance the hypothesis that the unexpected Chl *a* signal relates to the presence of picocyanobacteria found in deep anoxic waters and that better characterising these organisms could challenge our current understanding of the increase in fluorescence from 100 to 1000 m in the Black Sea.

To test our hypothesis, two sampling campaigns were planned to monitor the in situ profile of Chl *a*, oxygen concentration and other environmental parameters and to sample for quantifying *Synechococcus* sp. in the deep layer of the Black Sea. To demonstrate the relation between the “deep red fluorescence” and *Synechococcus* Chl *a*, we isolated and cultivated the *Synechococcus* spp. we recovered, characterized their genomes, constructed a phylogenetic tree and conducted a laboratory experiment to check their survival and behaviour in dark anoxic conditions and in the presence of light and oxygen.

## Methods

### Bio-Argo autonomous profiling floats

The Chl *a* profiles were measured by Bio-Argo autonomous profiling floats (model PROVORCTS-4 NUT, equipped with miniature and low-power sensors: Aanderaa oxygen optodes 4330 and WETLabs ECO-Triplet fluorometers) delivered to three marine sites: 7900591 in the Black Sea; 6901472 in the North Atlantic Ocean and 69001770 in the Mediterranean Sea. The Argo floats drift with deep currents at 1000 m and ascend every 10 days to the sea surface measuring continually various physical and biogeochemical properties of sea water. The data obtained in near real-time were processed by the Marine Optics and Remote Sensing lab in Villefranche and distributed via the Ocean Autonomous Observations web site (www.oao.obs-vlfr.fr).

### Sampling and chemical analyses

Seawater samples for chemical analyses were collected at St. 307 western gyre (43°10’N-29°00’E in DD 47.17N-29.00E) of the Black Sea on 25 June, 28 July 2015 and 22 June 2016 from 17 depths (from 0 to 1150 m) using a 12-Go Flo bottle CTD rosette sampler system (SBE-911 CTD). Selected samples for *Synechococcus* counting (at thermocline, at deep chlorophyll maximum (DCM), 250, 500, 750 and 1050 m) were fixed (1% formaldehyde, final concentration), kept at 4 °C in the dark and counted within 6 days. Temperature, salinity and pH were measured in situ with SBE-911 CTD system. Dissolved oxygen was measured by the Winkler technique [21]. Nutrients (phosphate, silicate, nitrate, nitrite and ammonia) were analysed using standard methods [22, 23]. Hydrogen sulphide was measured photometrically following Cline [24].

### *Synechococcus* sp. strain isolation

*Synechococcus* sp. strains BS55D and BS56D (hereafter referred to as BS55D and BS56D) were isolated on 25 June and 28 July 2015 from 750 m. During the field campaign samples for isolation and analyses were obtained by carefully washing the Niskin bottles of the Rosette sampler, following the consolidated protocol in order to avoid contamination [25]. The samples for the *Synechococcus* isolation (100 ml) were recovered from the sampling bottle and collected in a sterilised bottle. The samples were gravity filtered through a 3 μm polycarbonate membrane, in triplicate, and the membranes were placed in small sterilised culture vials with 3 ml of BG11 medium (prepared using deep Black Sea water, 0.2 µm filtered and sterilised) [26]. The vials were kept in a thermostat at 18–20 °C and low light (10–15 μmol photons m^−2^ s^−1^). To obtain cultures with a single picocyanobacteria strain, purification was performed using flow cytometric single-cell sorting with an InFlux V-GS flow cytometer (Becton Dickinson Inc., NJ, USA). A single autofluorescent cell was directly inoculated into a single well of a 96-well plate, each well containing 100 μl of BG11 medium [26]. It took almost 2 months to obtain a growth in the wells, visible as a pink film at the bottom of the small wells. The few volumes of the well were passed gradually to the larger cultured flasks. As a result, we successfully obtained monoclonal cultures from 750 m on both dates.

### Sequencing, assembly and annotation of BS55D and BS56D

DNA of pelleted strains was isolated with a Qiagen MagAttract Kit (Qiagen, Hilden, Germany), genomic 550-bp libraries were constructed with a KAPA Hyper Prep Kit (Kapa Biosystems, Wilmington, MA, USA) and sequenced on an Illumina MiSeq instrument, using a 500-cycle MiSeq Reagent v2 Kit (Illumina, San Diego, CA, USA). Paired-end reads were quality-filtered and trimmed with trimmomatic [27] and assembled with SPAdes [28] following meta, careful, only-assembler and default k-mer parameters. Gene prediction was conducted with PRODIGAL [29]. Annotation of CDS was done with BLAST [30] and RAST [31], KEGG KO [32, 33], COG [34] and TIGR [35] databases; tRNAs were detected with tRNAscan-SE [36], and 16S rRNA genes were determined with ssu-align [37]. Protein specific hits and domains were predicted with CDD-SPARCLE [38].

### Phylogenomics, synteny plots and PBS comparison of BS55D and BD56D

A maximum-likelihood phylogenetic tree with 259 universal markers and *Synechococcus* representatives from marine, brackish, euryhaline and freshwater habitats together with the novel strains was generated with a PhyloPhlAn tool [39]. Nine *Prochlorococcus* genomes were used to root the phylogeny. Synteny plots comparing Red Sea and Black Sea *Synechococcus* strains were made with BLASTN with > 70% identity hits and > 100 bp of alignment lengths. Average nucleotide identity (ANI) between different strains was also calculated as previously described [40]. The structure and similarity plots of the PBS gene cluster among marine, coastal and freshwater *Synechococcus* sp. were determined with TBLASTX with > 30% similarity hits and 150 bp alignment lengths.

### Laboratory experiment

The two strains BS55D and BS56D were genetically very similar, therefore only one (BS56D) of them was used to test survival and Chl *a* production under anoxic and dark conditions. In a 48-day experiment, a total of seven time points were selected for two treatments: the anoxic/dark and the oxic/light. The media (BG11 with Black Sea water, see paragraph on isolation) was made anoxic by stripping it overnight with a mixture of N_2_ (99.9%) and CO_2_ (0.1%). For the anoxic/dark treatment, 21 glass Winkler bottles (100-ml volume) were filled anoxically with media and a small concentrated inoculum (1 ml), and sealed with no headspace. For the oxic/light treatment, the same bottles were used but with a filter cap. The inoculated bottles were kept in a thermostatic chamber at 20 °C, 21 in the dark and 21 at 10 μmol photons m^−2^ s^−1^. Irradiance of the cool white fluorescent tubes (Slimline F72 T12 CW 55Watt) installed in the chamber were measured by QSL2110 underwater probe (Biospherical Instrument Inc.). At each sampling time point (3, 6, 13, 22, 27 and 48 days), three bottles were sacrificed for each treatment, the cells were counted, and Chl *a* and F_v_/F_m_ were measured.

### *Synechococcus* spp. enumeration

*Synechococcus* spp. from the field and during the laboratory experiment were counted with a Flow Cytometer‎ (Accuri C6, Becton Dickinson Inc., NJ, USA), equipped with a 20 mW 488 nm Solid State Blue Laser and a 14.7 mW 640 nm Diode Red Laser. Light scattering signals (forward and side light scatter named FSC-H and SSC-H, respectively), orange fluorescence (FL2-H channel = 585/40 nm) and red fluorescence (FL3-H channel > 670 nm and FL4-H channel 675/25) were used to distinguish and quantify *Synechococcus* cells. Threshold values were set at 7000 for FSC-H and 1500 for FL4-H channel and alternatively compared with 1000 for FL3-H and 2000 for FL4-H channel to obtain the best results. The density plots of FL2-H vs. FL3-H allowed for the optimal gating design and the quantification of the phycoerythrin-rich *Synechococcus* cells [41]. All data were acquired at a pre-set flow rate of 35 µL min^−1^, in order to keep the number of total events below 1000 per second. The BD Accuri C6 resident software (v. 1.0.264.21) was used for cytogram gating and data processing.

All samples were inspected at the epifluorescence microscope. A variable volume of water (2–10 ml) was filtered on white polycarbonate filters (Poretics, 0.2 -µm pore size) and observed with a Zeiss Axioplan microscope equipped with an HBO 100 W lamp, a Neofluar 100 × objective, 10  × ocular and 1.25 × additional magnification and filter sets for blue (BP450-490, FT510, LP520) and green light excitation (LP510-560, FT580, LP590).

### PhytoPAM Chl *a* and photosynthetic activity measurement

During the laboratory experiment, Chl *a* was measured by a Pulse-Amplitude-Modulation Phytoplankton Analyzer (PhytoPAM, Heinz Walz, GmbH, Effeltrich, Germany). PhytoPAM was equipped with the Optical Unit ED 101US/MP, the phyto ML (25 measuring LED in the 4 wavelenghs and 12 actinic LED 655 nm), and the phyto AL (37 actinic LED 655 nm) [42]. The µg Chl *a* ml^−1^ was measured at each time point of the experiment, using *Synechococcus* reference spectra and specific Chl *a* calibration as reference.

PhytoPAM was used to estimate the photosynthetic activity through the assessment of the effective quantum yield of energy conversion at the reaction centres of photosystem II (PSII) using saturation pulses. Therefore, the maximal quantum conversion efficiency of PSII as F_v_/F_m_ ratio in dark-adapted samples [43] was measured at each time point.

## Results

### Field measurements, *Synechococcus* enumeration and isolation

**Fig. 1:**
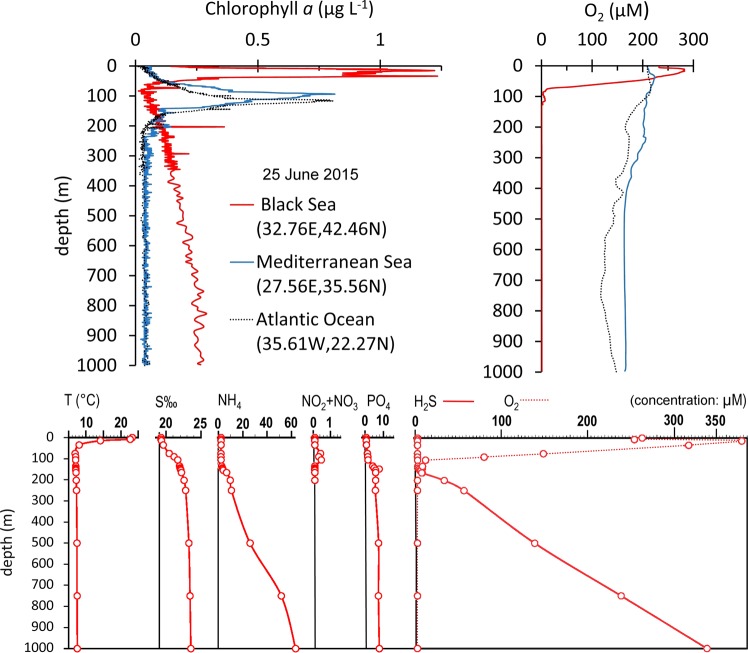
Chlorophyll *a* (upper left) and dissolved oxygen (upper right) concentration profiles measured by three Argo floats (model PROVORCTS-4 NUT). Dates and locations of the profiles are written in the legend. In the table, the chemical and physical parameters at St. 307 western gyre (43.17N-29.00E) of the Black Sea on 25 June 2015 are reported. The rapid decrease of O_2_ from 50 to 100 m (oxycline depth), reaching zero values at 100–200 m and keeping it up to 1000 m, confirms the anoxic deep zone of the Black Sea in comparison with the oxic deep zone of other oceans. Chemical analyses confirmed the presence of hydrogen sulphate (H_2_S) and ammonia (N–NH_4_) and the absence of nitrate from 100 to 1000 m

The Bio-Argo floats deployed on 25 June 2015 measured a Chl *a* profile with the pattern already observed since 2013 in the western gyre (43.17N-29.00E) of the Black Sea. We noted an increase of Chl *a* between 100 and 1000 m depth, reaching values of around 0.2–0.3 µg L^−1^, which were neither visible in similar profiles obtained on the same dates in the Mediterranean Sea nor the Atlantic Ocean (Fig. 1, upper left). Lower values of Chl *a* (3 ng Chl L^−1^) were measured at 1000 m with pigment extraction and HPLC measurements by other authors [44]. The in situ measurement of oxygen confirmed the anoxic condition of the Black Sea in comparison with the oxygenated Mediterranean Sea and Atlantic Ocean (Fig. 1, upper right).

**Fig. 2:**
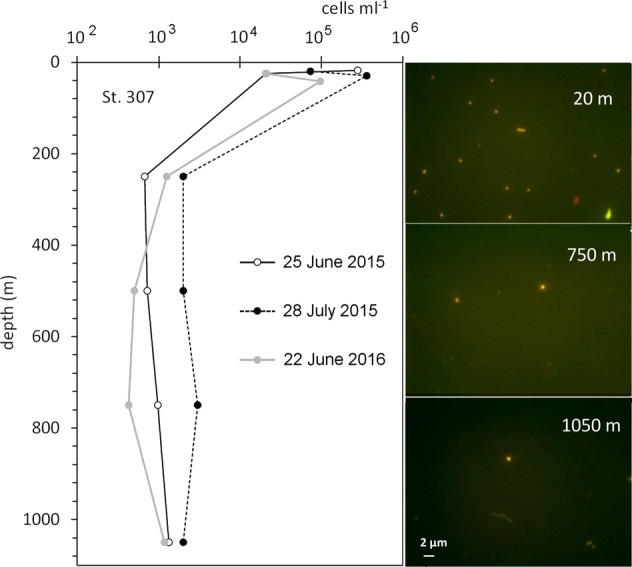
*Synechococcus* spp. abundance profiles in the western gyre of the Black Sea (St. 307: 43.17N-29.00E) performed during two cruises in 2015 and compared with a new profile obtained in summer 2016. On the right: examples of epifluorescence microscopy visualisation of *Synechococcus* spp. at three depths (Zeiss Axioplan, 1250 × , blue excitation: P450-490, FT510, LP520)

From the profiles, oxygen concentration was higher at 15 m (374 µM) and confirmed to be zero from around 200 to 1000 m, whereas H_2_S and NH_4_ increased progressively down to 1000 m where they reached 334 and 65 µM, respectively (Fig. 1, lower panels). The profiles of *Synechococcus* cell abundance in the three dates indicated the presence of 10^3^ cells ml^−1^ of phycoerythrin-rich picocyanobacteria of the genus *Synechococcus* from around 200 m to 1000 m, with the highest number (358 × 10^3^ cells^−1^ ml^−1^) found at DCM (Fig. 2). The observation at the microscope confirmed the presence of *Synechococcus* cells with high autofluorescence at depth (Fig. 2), and with a mean cell size of 0.60 ± 0.03 × 0.38 ± 0.02 µm at 20 m, 0.80 ± 0.09 × 0.69 ± 0.01 µm at 750 m.

From samples taken at 750 m on June and July 2015 in the western gyre of the Black Sea, we successfully isolated two *Synechococcus* sp. strains, BS55D and BS56D, confirming the presence of these prokaryotic cells in the deep mesopelagic water. Both strains, even if isolated in different months (June and July), have similar phenotype and genotype. They are composed by phycoerythrin-rich rod-shaped cells with similar size (BS55D: 1.30 × 0.69 µm and BS56D: 1.69 × 0.79 µm) (Fig. S1). The cultures are pink in colour and did not turn green after exposure to red light. The absorption spectra of the two strains are very similar to the main peak of phycoerythrin at 573 nm and of Chl *a* at 443 and 682 nm, with a very reduced peak of allophycocyanin at 363 nm (Fig. S2).

### Survival experiment and Chl *a* production in anoxic dark conditions

**Fig. 3:**
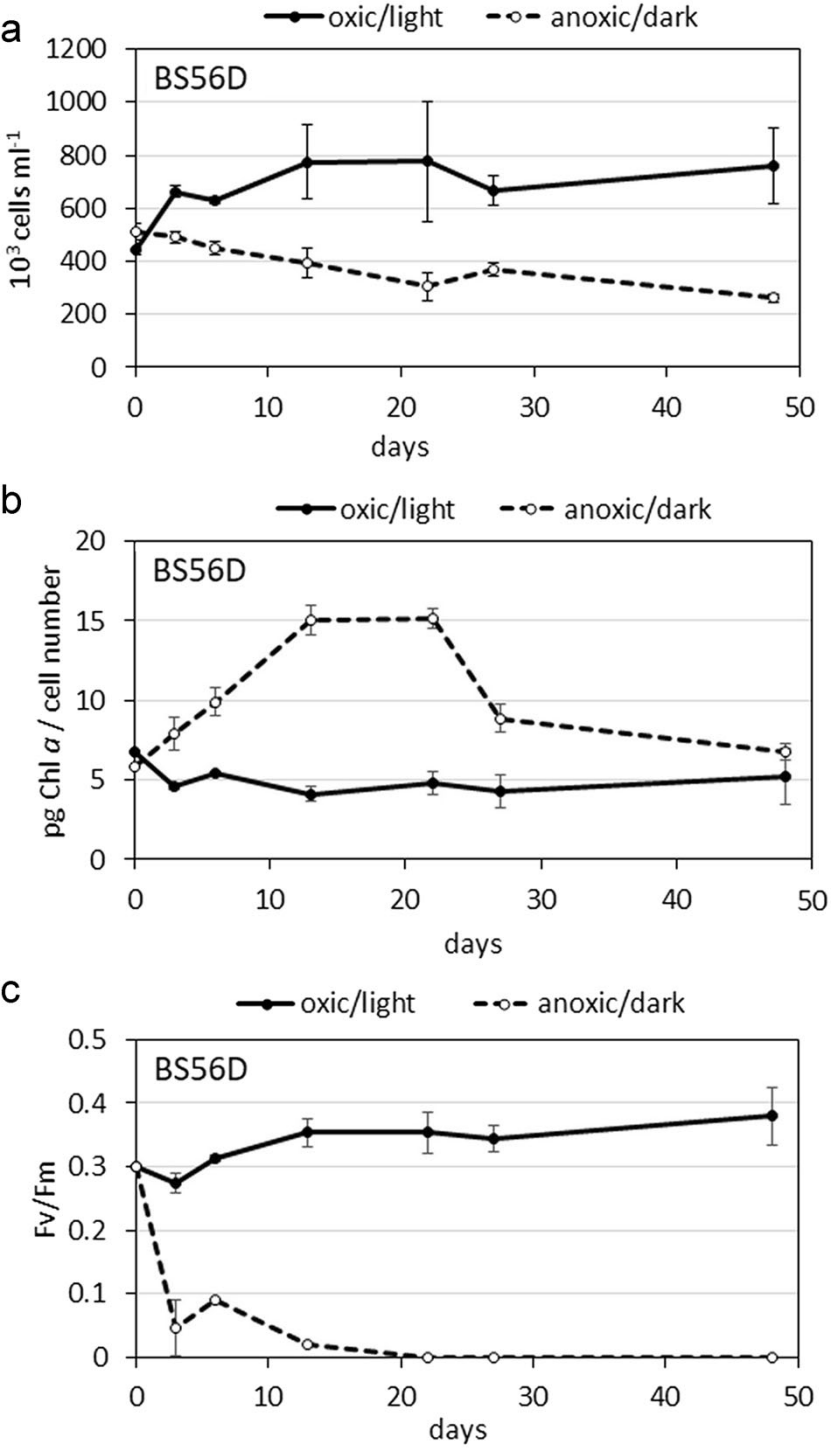
Experiment of *Synechococcus* BS56D growth in oxic/light and anoxic/dark conditions during 48 days. **a** Number of *Synechococcus* (10^3^ cells ml^−1^), **b** Chlorophyll *a* cell content (pg Chl *a* cell^−1^), **c** Photosynthetic activity of PSII (Fv/Fm). Statistical analysis (*t* test) has shown significant differences between the two treatment (**a**: *p* = 0.0103, **b**: *p* = 0.0245, **c**: *p* = 0.0018)

During the 48-day laboratory incubation, the strain BS56D had a positive growth rate (k = 0.011 d^−1^) in the oxic/light conditions and a negative growth rate (k = −0.014 d^−1^) in anoxic/dark conditions (Fig. 3a). During the first week, the cells survived quite well also in the anoxic/dark conditions, even if not growing in number, and after 13 days they decreased by 23% from the initial number but they presented high cellular Chl *a* concentrations (pg Chl *a* cell^−1^) reaching 15 pg Chl *a* cell^−1^ and maintaining this concentration from day 13 to day 22 (Fig. 3b). The cellular concentration of Chl *a* in oxic/light conditions were significantly lower (*t* test: *p*-value 0.0245) than those observed in anoxic/dark, which doubled in the first 13 days of incubation. Conversely, in oxic/light conditions, the cell number increased, and indicated photosynthetic activity of the PSII, lacking in the anoxic/dark treatment (Fv/Fm were 0.35 in oxic/light compared with 0.0 value for the anoxic/dark after 13 days of cultivation) (Fig. 3c).

### Genomic features of BS55D and BS56D

Here we present the main characteristics of the genomes of BS55D and BS56D, the novel strains isolated from 750 m at St. 307 western gyre (43.17N-29.00E) of the Black Sea. Their genomes have a total size of 2303823 and 2235215 bp and a GC content of 61.23 and 61.61, for BS56D and BS55D, respectively. An overview of selected metabolic pathways inferred from *Synechococcus* sp. strains BS55D and BS56D genomes is presented in Table S1.

#### Chl *a* biosynthesis

We found both enzymes involved in some steps of the aerobic, micro-oxic and anaerobic pathways of tetrapyrrole biosynthesis in BS55D and BS56D. In Cyanobacteria, the coproporphyrinogen III oxidation to produce protoporphyrinogen IX containing the vinyl groups of the tetrapyrrolles is catalysed by an oxygen-dependent oxidase (hemF) while an oxygen-independent oxidase (hemN) is responsible for the anaerobic pathway of the same protoporphyrinogen IX biosynthesis. In the two strains, we found both variants. Another important step of the production of Chl *a* is the Mg-protoporphyrin IX monomethyl ester cyclization or MPE, which involves an oxygen-dependent MPE cyclase (AcsF or ChlE) in the aerobic pathway and a radical SAM family variant (BchE) for the anaerobic cyclization. BLAST searches among the entire cyanobacterial phylum have shown that a very limited number of them (as *Cyanothece* spp. PCC 7425 and PCC 7822) contain both variants, although we also observed the presence of BchE and ChlE in the two picocyanobacterial strains analysed. The next-to-last step on the Chl *a* biosynthesis is the protochlorophyllide reduction to chlorophyllide *a*, which is catalysed by a stereo-specific hydrogenation that has a light-dependent single polypeptide oxidoreductase variant (LPOR) and a light-independent three subunits one (DPOR). Two LPOR and one DPOR were detected in both Black Sea strains.

#### Putative anaerobic pathways

As depicted by their genomic analysis, BS55D and BS56D are potentially capable of fermenting intracellular polyglucose to lactate. We recovered the D-lactate dehydrogenase that catalyses the conversion of lactate to pyruvate and vice versa with the production of NAD+. Furthermore, these two organisms could be potentially capable of heterofermentation yielding lactate and acetate (no ethanol), as the phosphoketaolase D-xylulose-5-phosphate D-glyceraldehyde-3-phosphate-lyase was also detected. We also observed the presence of the pathway involved in the fermentation of pyruvate to acetoin via acetolactate synthase (*alsC, budB*) and alpha-acetolactate decarboxylase (*alsD, budA*), although we did not identify the butanediol dehydrogenase involved in the reversible conversion of acetoin into 2,3-butanediol.

#### Nitrogen metabolism

With regard to the nitrogen metabolism, we detected the presence of cyanate hydratase (hydrolysis of cyanate to ammonia and CO_2_), urease activity (*ure* cluster), ammonia transporters (*amt*) and assimilation enzymes (GOGAT) and nitrate/nitrite ammonification pathways (nitrate assimilation through *nrtP* and *narB*; nitrite assimilation through *focA*, *nirA* and *corA*), which suggests that these two Black Sea strains have a wide set of tools to incorporate different sources of N in the cells, all of them converging to ammonia. It should be noted that in the mesopelagic Black Sea, ammonia is the prevalent form of nitrogen and the strains BS55D and BS56D, as other cyanobacteria, have the molecular tools to use ammonia.

#### Heterotrophy genes

Our strains from Black Sea were also explored at the genomic level in order to see their potential heterotrophic ability. BS55D and BS56D encode transporters for polar amino acids and sugars (ABC-sugar transporter *ugpAE*) and peptides (ABC-type dipeptide transport system). As expected, we found evidence for glucose uptake with transporters, such as a GPH family sugar transporter (*melB*) and an *oprB* porin/glucose porter and degradation genes such as glucokinase and glucose-6-phosphate-1-dehydrogenase. We also detected polar amino acid permeases genes (*bzt* and *potE*) and their respective substrate-binding proteins. It must be noted that our strains also present a sodium alanine/glycine symporter together with the degradation enzyme ald, alanine dehydrogenase. Finally, we also found a glutamate/leucine dehydrogenase gene (*gdhA*), but we did not detect any specific symporter sodium aspartate/glutamate. Altogether, the presence of these genes exemplifies the putative ability of our strains to take up organic compounds, which will have to be verified by appropriate laboratory experiments.

### Phylogenomics, synteny and PBS organisation of Black Sea *Synechococcus*

**Fig. 4:**
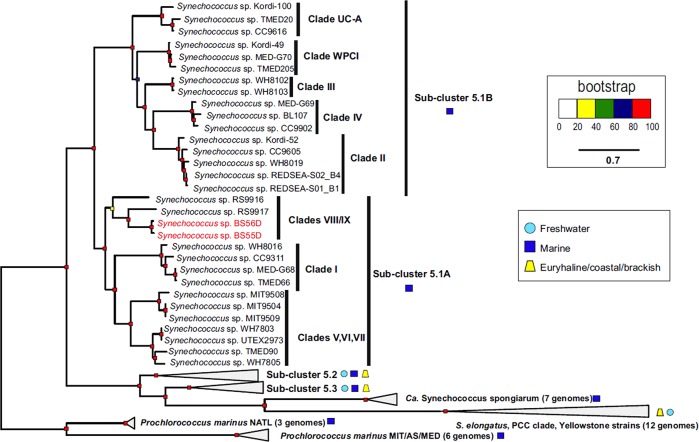
Phylogenomics of *Synechococcus* BS55D and BS56D spp. Maximum-likelihood phylogenomic tree with representatives from marine, brackish, euryhaline and freshwater habitats together with the novel strains. A total of 259 universal genes were used out of the 400 markers from PhyloPhlAn tool. Nine *Prochlorococcus* genomes were used to root the phylogeny

In order to know to which clade to ascribe the novel strains, we performed a maximum-likelihood phylogenetic tree (Fig. 4) of 239 conserved genes (extracted with the PhyloPlan tool) comprising representatives from the marine sub-clusters 5.1 A/B, 5.2, 5.3 and freshwater *Synechococcus*. We used nine genomes of *Prochlorococcus* to root the phylogeny. As could be expected and due to its marine origin, BS55D and BS56D strains fell inside the 5.1 sub-cluster close to the clades VIII/IX comprising Red Sea strains RS9916 and RS9917. The relations among Red Sea strains *Synechococcus* sp. RS9917/RS9916 and Black Sea strain *Synechococcus* sp. BS56D and BS55D are well represented in the synteny plots (Fig. S3). The ANI between Red Sea and Black Sea strains showed that they were closest to the RS9917 representative of clade VIII.

**Fig. 5:**
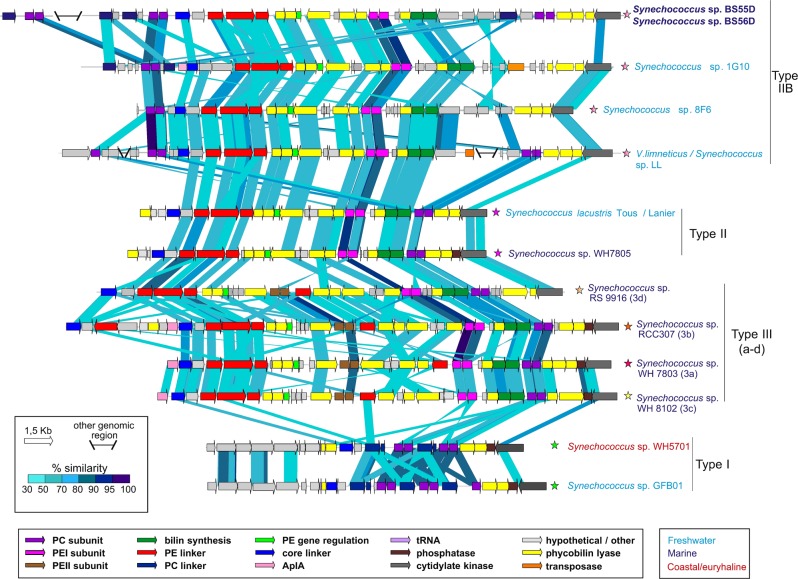
Structure and similarity of the PBS gene cluster among marine, coastal and freshwater *Synechococcus* spp. including the new BS55D and BS56D. PBS types I, II, III (a–d) and IIB. Comparison made with TBLASTX with > 30 % similarity hits and 150 bp alignment lengths. PC: Phycocyanin; PE: Phycoerythrin; AplA: Allophycoyanin-like protein; Phycobilin lyases: CpeY, CpeZ, CpeF, CpeS, CpeT, CpeU, RpcG, RpcE, RpcE, RpcF, RpcT; Bilin synthesis: pebA, pebB. Origin of each genome is colour coded. Star symbols represent the pigment colour of each strain

BS55D and BS56D strains contain the novel pigment type IIB (Fig. 5) reported in some freshwater strains [45]. In addition, a total of six phycocyanin subunits were found, four of them inside the PBS operon and two more phycocyanins elsewhere in the genome. The presence of such amount of phycocyanin subunits has been reported in the green pigmented picocyanobacteria exposed to more light [46], but never in phycoerythrin-containing cells.

## Discussion

The in situ profiles in the western gyre of the Black Sea demonstrate the occurrence of deep red fluorescence in its meso- and bathypelagic waters. Microscopy and flow-cytometric analyses of samples along the whole profile down to 1000 m showed highly fluorescent cells of PE-rich picocyanobacteria belonging to the genus *Synechococcus* (Fig. 2, Fig. S1). The detection of *Synechococcus* cells at depth (up to 10^3^ cells ml^−1^) led us to formulate the hypothesis that the Chl *a* increase often called “deep red fluorescence” is linked to a chromophore/fluorophore originating in this PE-rich photoautotrophic cyanobacteria, as early investigations of the phenomenon had already speculated [1, 13]. At present, we are not able to unambiguously quantify the contribution of the living *Synechococcus* cells to the total Chl *a* fluorescence signal measured by the Bio-Argo float. But, to our knowledge, this was the first time the presence of *Synechococcus* cells living in the harsh conditions of the mesopelagic Black Sea water has been convincingly demonstrated, lending more valence to the hypothesised connection between the signal and cyanobacteria and opening to novel interpretations of the “deep red fluorescence” phenomenon.

The results of our study begin to provide experimental evidence that support this hypothesis by characterizing two *Synechococcus* sp. strains isolated from the mesopelagic realm of the Black Sea and demonstrating their ability to survive both in anoxic/dark and oxic/light conditions, and to accumulate Chl *a* in the former.

While the recovery of *Synechococcus* in the anoxic/dark conditions found in the deep Black Sea was novel and unexpected, it is consistent with the fact that different oxygen concentrations have led to the development of aerobic, micro-oxic and anerobic variants of the enzymes involved in the tetrapyrrole (chlorophylls, hemes and bilins) biosynthesis in Cyanobacteria [47]. Indeed, *Synechococcus* sp. PCC 7002, *Leptolyngbya boryana*, *Cyanothece* sp. PCC 7425, *Anabaena variabilis*, *Synechocystis* sp. PCC 6803 and other Cyanobacteria contain the vast majority of the aerobic, micro-oxic and anaerobic pathways for tetrapyrrole biosynthesis [47]. Our findings illuminate the metabolic pathways encoded in the genome of our strains, and shed light on their relation with the observed environmental conditions and Chl *a* signal.

The genomes of BS55D and BS56D contained genes encoding two variants of the enzymes catalysing the aerobic and anaerobic pathways for tetrapyrrole biosynthesis. At present, our characterisation of BS56D, used in the experiment, allows us to formulate two possible hypotheses to understand the Chl *a* accumulation in the anoxic/dark condition. The first considers it a strategy for the fast recovery of photosynthetic activity upon dark–light transition, also demonstrated by the fact that the *Synechococcus* strains which lived in anoxic/dark conditions at 750 m in the Black Sea were successfully cultivated and were able to reproduce when exposed to oxygen and light. The capacity of conducting Chl *a* biosynthesis in the absence of light is a characteristic of cyanobacteria and involves a series of processes: the reduction of protochlorophyllide reductase, the expression of photosynthesis genes and the assembly of photosystems [48, 49]. Though PSI and PSII are inactive in the dark, some Chl *a* can be stored freely, i.e., not associated to the photosynthetic systems, thus making them able to quickly react to light in case of exposure. From studies on the chlorophyll cycle, it was revealed that chlorophyll synthesis is tightly coordinated for the construction of the photosynthetic apparatus so that the accumulation of Chl *a* in BS56D can be considered a potential regulator of photosynthetic machinery [50], and interpreted as a chance for a new photosynthetic resurrection. The successful isolation of BS55D and BS56D from deep, anoxic/dark conditions, and their growth in oxic/light conditions is evidence of the high adaptability of these strains.

Whole-genome sequencing allowed us to characterise the metabolic machinery of the strains, which seems to retain enduring ancestral pathways and resort to them to adapt to adverse environments. For example, the DPOR pathway we found in these strains evolved from nitrogenase-related genes [51] and was the sole protochlorophyllide reductase for Chl *a* biosynthesis in ancestral cyanobacteria [52]. The activity of the light-independent variant for Chl *a* biosynthesis might have a role in explaining the relation between picocyanobacteria and the Chl *a* increase in deep dark waters.

We have strong evidence that BS55D and BS56D could perform different kinds of fermentation, as other cyanobacteria do [53, 54]. We recovered the D-lactate dehydrogenase and specific lyases to perform heterofermentation, and we also detected the presence of a pathway involved in the fermentation of pyruvate to acetoin via acetolactate synthase subunits and alpha-acetolactate decarboxylase. It must be noted that D-lactate dehydrogenase was only detected in one other *Synechococcus* strain (RS9917, isolated from Red Sea). The apparent absence of this enzyme in marine *Synechococcus* (except for the strain RS9917) is surprising and is a distinctive feature of our Black Sea ecotype, which survived well in anoxic conditions for a relatively large period of time. The next step will be to ensure the true activity of these potential pathways reconstructed by genome analysis.

With regard to the nitrogen metabolism, we observed cyanate hydratase (hydrolysis of cyanate to ammonia and CO_2_), urease activity, ammonia transporters, assimilation enzymes and nitrate/nitrite ammonification pathways. The presence of genes involved in all these metabolic pathways suggests that BS55D and BS56D have a wide set of tools to incorporate different sources of N into the cells, all of them converging to ammonia, as previously reported in other marine *Synechococcus* strains [55]. Nonetheless, it must be noted that the strain RS9917 (isolated from Red Sea), the closest one relative to the Black Sea species, lacks the nitrate assimilation genes [55]. Common picocyanobacterial pathways of PO_4_ and H_2_S assimilation were detected in both *Synechococcus* strains. It is likely that the increasing concentrations with depth of H_2_S, PO_4_ and NH_4_ observed in the profiles of the Black Sea, create conditions that are viable if fermentation and other anoxygenic pathways are activated.

An alternative interpretation of our results relies on the ability of BS55D and BS56D to complement their photoautotrophic metabolism with heterotrophic pathways [56]. Both BS55D and BS56D encode transporters for polar amino acids, sugars and peptides, similar to those found in 67 reference picocyanobacteria [56]. As expected, we found evidence for glucose uptake with transporters and the presence of permeases and dehydrogenases. Altogether, the presence of the respective genes confirms the putative ability of our strains to take up organic compounds. We are aware that the presence of these genes does not necessarily mean that these are actively used, but it indicates the potential ability to carry out a metabolic pathway.

Another feature that was observed in our Black Sea strains was the presence of the novel pigment type IIB described for the Baltic Sea [57], a feature previously observed in freshwater picocyanobacteria and brackish representatives [45], but not observed in marine ecotypes possessing types I, II and III (including chromatic adaptors) [55, 58]. This fact hints at a possible dominance of type IIB pigmentation in the Black Sea picocyanobacteria and expands horizons on the PBS evolution and subunits transference among Cyanobacteria. In addition, in BS55D and BS56D a total of six phycocyanin subunits have been found, four of them inside the PBS operon and two phycocyanins not located inside this cluster but elsewhere in the genome. The presence of such a high amount of phycocyanin subunits was reported in green pigmented picocyanobacteria that are more exposed to light [59], but never in phycoerythrin-containing cells. This gene complement could enable the Black Sea strains to synthesise PBS rods containing phycoerythrin and multiple discs of phycocyanin (considering up to six PC subunits). This may result in unique light absorption features of the cells along the Black Sea high/low light profile, serving as light energy transfer to Chl *a* in the PSII, while in the dark anoxic basin of the Black Sea their functionality remains unknown.

Strains, BS55D and BS56D, fall inside the 5.1B sub-cluster, being close to clades VIII/IX containing Red Sea strains, RS9916 and RS9917 [60]. Specifically, they are closer to the RS9917 representative (clade VIII), although they form a clearly distinct branch, opening new perspectives for a novel clade originating from the Black Sea, with the ability to live in the dark anoxic layer of that sea and potentially in the OMZ of the subtropical oceans [9, 12].

The viable stock of BS55D and BS56D strains in the Black Sea’s bathypelagic waters retains photosynthetic ability upon re-exposure to light, and our experiment demonstrates that it accumulates Chl *a* in dark hypoxic habitats, potentially in order to facilitate this process. While calling for further research on these dark, anoxic habitats and their cyanobacterial populations, these findings seem to lend support to earlier speculations that associated cyanobacterial metabolisms to the Chl *a*-like signal known as “deep red fluorescence” [11]. The presence and concentration of *Synechococcus* spp. along the profile; the genomes and metabolic machinery of two strains of *Synechococcus* isolated from the deep water; their phylogenetic position; together with the accumulation of Chl *a,* we tested and the strains’ resilience in the dark, all provide strong evidence that links the still poorly understood signal with the presence of *Synechococcus*. This, in turn, challenges the understanding of the Chl *a* signal at depth as an artefact of the measurement technique, and its underlying assumption that Chl *a* has no significant role in dark anoxic waters. Given the substantial contribution of the ocean dark zone to global nutrient dynamics, further exploring the role of picocyanobacteria we suggest here promises to shed more light on the processes shaping these important dark environments and their global impact.

In short, our results demonstrate the occurrence of “deep red fluorescence” in the meso- and bathypelagic waters of the Black Sea. They also confirm the presence of *Synechococcus* spp. in coincidence with this Chl *a* signal, and validate the ability of strain BS56D to survive in anoxic/dark deep waters. In addition, they show the accumulation of Chl *a* by strain BS56D under these environmental conditions. As such, they corroborate the hypothesized relation between the Chl *a* increase at depth and the presence of *Synechococcus* spp. in the mesopelagic and bathypelagic realm of the Black Sea, up to now considered unsuitable for the survival of photoautotrophic cells [61], while also calling for more research on this process.

## Supplementary information

Supplementary Table and Figures

## Data Availability

The Black Sea *Synechococcus* genomes have been deposited to GenBank-NCBI under bioproject PRJNA419515. *Synechococcus* sp. BS55D is codified by biosample SAMN08057241 and Genbank accession number PHQT00000000. *Synechococcus* sp. BS56D is codified by biosample SAMN08057242 and Genbank accession number PHQU00000000.

## References

[1] Broenkow WW, Yuen MA, Yarbrough MA (1992). VERTEX: biological implications of total attenuation and chlorophyll and phycoerythrin fluorescence distributions along a 2000 m deep section in the Gulf of Alaska. Deep Sea Res Part A.

[2] Konovalov SK, Murray JW, Luther GW (2005). Basic processes of Black SeaBiogeochemistry. Oceanography.

[3] Murray JW, Top Z, Özsoy E (1991). Hydrographic properties and ventilation of the Black Sea. Deep Sea Res Part A Ocean Res Pap.

[4] Stanev E, He Y, Grayek S, Boetius A (2013). Oxygen dynamics in the Black Sea as seen by Argo profiling floats. Geophys Res Lett.

[5] Stanev EV, He Y, Staneva J, Yakushev E (2014). Mixing in the Black Sea detected from the temporal and spatial variability of oxygen and sulfide–Argo float observations and numerical modelling. Biogeosciences.

[6] Kucuksezgin F, Pazı I (2003). Vertical structure of the chemical properties of western Black Sea. Indian J Mar Sci.

[7] Zaitsev Y, Mamaev V (1997). Marine biological diversity in the Black Sea. A study of change and decline. United Nation Publications. GEF Black Sea Environ Ser.

[8] Anderson JJ (1982). The nitrite-oxygen interface at the top of the oxygen minimum zone in the eastern tropical North Pacific. Deep Sea Res Part A.

[9] Broenkow WW, Lewitus AJ, Yarbrough MA, Krenz RT (1983). Particle fluorescence and bioluminescence distributions in the eastern tropical Pacific. Nature.

[10] Broenkow WW, Lewitus AJ, Yarbrough MA (1985). Spectral observations of pigment fluorescence in intermediate depth waters of the North Pacific. J Mar Res.

[11] Breves W, Heuermann R, Reuter R (2003). Enhanced red fluorescence emission in the oxygen minimum zone of the Arabian Sea. Ocean Dynam.

[12] Röttgers R, Koch BP (2012). Spectroscopic detection of a ubiquitous dissolved pigment degradation product in subsurface waters of the global ocean. Biogeosciences.

[13] Zhao Z, Gonsior M, Luek J, Timko S, Ianiri H, Hertkorn N (2017). Picocyanobacteria and deep-ocean fluorescent dissolved organic matter share similar optical properties. Nat Comm.

[14] Organelli E, Barbieux M, Claustre H, Schmechtig C, Poteau A, Bricaud A (2017). Two databases derived from BGC-Argo float measurements for marine biogeochemical and bio-optical applications. Earth Syst Sci Data.

[15] Xing X, Claustre H, Boss E, Roesler C, Organelli E, Poteau A (2017). Correction of profiles of in‐situ chlorophyll fluorometry for the contribution of fluorescence originating from non‐algal matter. Limnol Oceanogr-Meth.

[16] Callieri C, Cronberg G, Stockner J, Whitton B (2012). Freshwater Picocyanobacteria: single cells, microcolonies and colonial forms. Ecology of Cyanobacteria II: their diversity in time and space.

[17] Scanlan DJ, Whitton B (2012). Marine Picocyanobacteria. Ecology of Cyanobacteria II: their diversity in time and space.

[18] Sohrin R, Isaji M, Obara Y, Agostini S, Suzuki Y, Hiroe Y (2011). Distribution of *Synechococcus* in the dark ocean. Aquat Micro Ecol.

[19] Callieri C (2017). *Synechococcus* plasticity under environmental changes. FEMS Microbiol Lett.

[20] Cottrell MT, Kirchman DL (2009). Photoheterotrophic microbes in the Arctic Ocean in summer and winter. Appl Environ Microbiol.

[21] Hansen HP. Determination of oxygen. In: Grasshoff K, Kremling K, Ehrhardt m (editors) Methods of seawater analysis, 3rd edn. Germany: Wiley-VCH Verlag GmbH; 2007. p. 75–89.

[22] Grasshoff K, Kremling K, Ehrhardt M. Methods of seawater analysis. Germany: Wiley-VCH Verlag GmbH; 1999. 632 p.

[23] Solorzano L (1969). Determination of ammonia in natural waters by the phenol hypochlorite method. Limnol Oceanogr.

[24] Cline JD (1969). Spectrophotometric determination of hydrogen sulphide in natural waters. Limnol Oceanogr.

[25] Warren GJ (1996). Field sampling using Rosette sampler.

[26] Callieri C, Coci M, Corno G, Macek M, Modenutti B, Balseiro E (2013). Phylogenetic diversity of nonmarine picocyanobacteria. FEMS Microbiol Ecol.

[27] Bolger AM, Lohse M, Usadel B (2014). Trimmomatic: a flexible trimmer for Illumina sequence data. Bioinformatics.

[28] Bankevich A, Nurk S, Antipov D, Gurevich A, Dvorkin M, Kulikov AS (2012). SPAdes: a new genome assembly algorithm and its applications to single-cell sequencing. J Comput Biol.

[29] Hyatt D, Chen GL, Locascio PF, Larimer FW, Hauser LJ (2010). Prodigal: prokaryotic gene recognition and translation initiation site identification. BMC Bioinforma.

[30] Altschul SF, Madden LT, Shaffer A, Zhang J, Zhang Z (1997). Gapped BLAST and PSI-BLAST: a new generation of protein database search programs. Nucleic Acids Res.

[31] Overbeek R, Olson R, Pusch GD, Olsen GJ, Davis JJ, Disz T (2014). The SEED and the rapid annotation of microbial genomes using subsystems technology (RAST). Nucleic Acids Res.

[32] Kanehisa M, Goto S, Kawashima S, Okuno Y, Hattori M (2004). The KEGG resource for deciphering the genome. Nucleic Acids Res.

[33] Kanehisa M, Sato Y, Morishima K (2016). BlastKOALA and GhostKOALA: KEGG tools for functional characterization of genome and metagenome sequences. J Mol Biol.

[34] Tatusov RL, Natale DA, Garkavtsev IV, Tatusova TA, Shankavaram UT, Rao BS (2001). The COG database: new developments in phylogenetic classification of proteins from complete genomes. Nucleic Acids Res.

[35] Haft DH, Brendan JL, Richardson DL, Yang F, Eisen JA, Paulsen IT (2001). TIGRFAMs: a protein family resource for the functional identification of proteins. Nucleic Acids Res.

[36] Lowe TM, Eddy SR (1997). tRNAscan-SE: a program for improved detection of transfer RNA genes in genomic sequence. Nucleic Acids Res.

[37] Nawrocki EP, Eddy SR. Ssu-align: a tool for structural alignment of SSU rRNA sequences. http://eddylab.org/software/ssu-align/, 2010.

[38] Marchler-Bauer A, Bo Y, Han L, He J, Lanczycki CJ, Lu S (2016). CDD/SPARCLE: functional classification of proteins via subfamily domain architectures. Nucleic Acids Res.

[39] Segata N, Börnigen D, Morgan XC, Huttenhower C (2013). PhyloPhlAn is a new method for improved phylogenetic and taxonomic placement of microbes. Nat Commun.

[40] Konstantinidis KT, Tiedje JM (2005). Genomic insights that advance the species definition for prokaryotes. P Natl Acad Sci USA.

[41] Callieri C, Amalfitano S, Corno G, Bertoni R (2016). Grazing-induced *Synechococcus* microcolony formation: experimental insights from two freshwater phylotypes. FEMS Microbiol Ecol.

[42] Schreiber U, Bilger W, Schliwa U (1986). Continuous recording of photochemical and non-photochemical chlorophyll fluorescence quenching with a new type of modulation fluorometer. Photosynth Res.

[43] Genty B, Briantais JM, Baker NR (1989). The relationship between the quantum yield of photosynthetic electron transport and quenching of chlorophyll fluorescence. BBA-Gen Subj.

[44] Repeta D, Simpson D (1991). The distribution and recycling of chlorophyll, bacteriochlorophyll and carotenoids in the Black Sea. Deep-Sea Res.

[45] Sanchez-Baracaldo P, Bianchini G, Di Cesare A, Callieri C, Chrismas NAM. Insights into the evolution of picocyanobacteria and phycoerythrin genes (*mpeBA* and *cpeBA*). Front Microbiol. 2019;10:45. 10.3389/fmicb.2019.00045 PMC6363710 30761097

[46] Cabello-Yeves PJ, Picazo A, Camacho A, Callieri C, Rosselli R, Roda-Garcia JJ (2018). Ecological and genomic features of two widespread freshwater picocyanobacteria. Environ Microbiol.

[47] Fujita Y, Tsujimoto R, Aoki R (2015). Evolutionary aspects and regulation of tetrapyrrole biosynthesis in cyanobacteria under aerobic and anaerobic environments. Life.

[48] Kada S, Koike H, Satoh K, Hase T, Fujita Y (2003). Arrest of chlorophyll synthesis and differential decrease of Photosystems I and II in a cyanobacterial mutant lacking light-independent protochlorophyllide reductase. Plant Mol Biol.

[49] Aoki R, Hiraide Y, Yamakawa H, Fujita Y (2014). A novel “oxygen-induced” greening process in a cyanobacterial mutant lacking the transcriptional activator ChlR involved in low-oxygen adaptation to tetrapyrrole biosynthesis. J Biol Chem.

[50] Tanaka R, Tanaka A (2011). Chlorophyll cycle regulates the construction and destruction of the light-harvesting complexes. Biochim Biophys Acta.

[51] Armstrong GA (1998). Greening in the dark: light-independent chlorophyll biosynthesis from anoxygenic photosynthetic bacteria to gymnosperms. J Photoch Photobio B.

[52] Blankenship RE (2001). Molecular evidence for the evolution of photosynthesis. Trends Plant Sci.

[53] Oren A, Shilo M (1979). Anaerobic heterotrophic dark metabolism in the cyanobacterium *Oscillatoria limnetica*: sulfur respiration and lactate fermentation. Arch Microbiol.

[54] Stal LJ, Moezelaar R (1997). Fermentation in cyanobacteria. FEMS Microbiol Rev.

[55] Scanlan DJ, Ostrowki M, Mazard S, Dufresne A, Garczarek L, Hess WR (2009). Ecological genomics of marine picocyanobacteria. Microbiol Mol Biol R.

[56] Yelton AP, Acinas SG, Sunagawa S, Bork P, Pedrós-Alió C, Chisholm SW (2016). Global genetic capacity for mixotrophy in marine picocyanobacteria. ISME J.

[57] Larsson J, Celepli N, Ininbergs K, Dupont CL, Yooseph S, Bergman B (2014). Picocyanobacteria containing a novel pigment gene cluster dominate the brackish water Baltic Sea. ISME J.

[58] Dufresne A, Ostrowski M, Scanlan DJ, Garczarek L, Mazard S, Palenik B (2008). Unraveling the genomic mosaic of a ubiquitous genus of marine cyanobacteria. Genome Biol.

[59] Six C, Thomas JC, Garczarek L, Ostrowski M, Dufresne A, Blot N (2007). Diversity and evolution of phycobilisomes in marine *Synechococcus* spp.: a comparative genomics study. Genome Biol.

[60] Fuller NJ, Marie D, Partensky F, Vaulot D, Post AF, Scanlan DJ (2003). Clade-specific 16S ribosomal DNA oligonucleotides reveal the predominance of a single marine *Synechococcus* clade throughout a stratified water column in the Red Sea. Appl Environ Microbiol.

[61] Zaitsev Y (2008). An Introduction to the Black Sea Ecology.

