# Supplementary material for: The mesopelagic anoxic Black Sea as an unexpected habitat for Synechococcus challenges our understanding of global “deep red fluorescence”
Source: ISME J. 2019 Feb 28;13(7):1676–87. doi: 10.1038/s41396-019-0378-z (PMC6776005; doi:10.1038/s41396-019-0378-z)
Supplement: Supplementary file 1 — Supplementary Table and Figures [file 41396_2019_378_MOESM1_ESM.docx]

**Table S1.** Overview of selected metabolic pathways inferred from the genome of *Synechococcus* strains BS55D and BS56D. Putative anaerobic pathways were added (fermentation and chlorophyll biosynthesis). Identity percentages and query coverages with closest *Synechococcus* relatives were determined with BLASTP.

| Protein/enzyme | Metabolic pathway | closest genome | % identity (BLASTP) | Query coverage |
| --- | --- | --- | --- | --- |
| D-lactate dehydrogenase ldhA | Lactate and acetate Heterofermentation | *Synechococcus* sp. RS9917 | 86 | 99 |
| Xylulose-5-phosphate phoshphoketolase/Fructose-6-phosphate phosphoketolase xfp xpk |  | *Synechococcus* sp. RS9917 | 93 | 99 |
| Acetate Kinase ackA |  | *Synechococcus* sp. RS9917 | 81 | 98 |
| Acetolactate synthase large subunit ilvB ilvG ilvI | Acetoin synthesis | *Synechococcus* sp. RS9917 | 91 | 99 |
| Acetolactate synthase small subunit ilvH ilvN |  | *Synechococcus* sp. RS9917 | 96 | 99 |
| Acetolactate synthase catabolic alsC budB |  | *Synechococcus* sp. CB0101 | 73 | 99 |
| Alpha-acetolactate decarboxylase alsD budA aldC |  | *Synechococcus* sp. CB0101 | 71 | 95 |
| Cyanate hydratase CynS | Cyanate hydrolysis | *Synechococcus* sp. RS9916 | 73 | 96 |
| Nitrite reductase nirA | Nitrate and nitrite ammonification | *Synechococcus* sp. RS9917 | 87 | 98 |
| Nitrite transporter focA |  | *Synechococcus* sp. CB0205 | 94 | 93 |
| Nitrite transporter CorA |  | *Synechococcus* sp. RS9917 | 68 | 98 |
| Nitrate transporter nrtP |  | Synechococcus sp. CB0101 | 93 | 99 |
| Assimilatory nitrate reductase narB |  | *Synechococcus* sp. MIT S9508 | 82 | 99 |
| Light-independent protochlorophyllide reductase subunit N chlN | Tetrapyrrole, chlorophyll biosynthesis | *Synechococcus* sp. RS9916 | 88 | 98 |
| Light-independent protochlorophyllide reductase subunit B chlB |  | *Synechococcus* sp. RS9916 | 85 | 100 |
| Light-independent protochlorophyllide reductase subunit L chlL |  | *Synechococcus* sp. WH8109 | 94 | 100 |
| Light-dependent Protochlorophyllide reductase por | Tetrapyrrole, chlorophyll biosynthesis | *Synechococcus* sp. RS9917 | 90 | 93 |
| Aerobic oxygen-dependent MPE cyclase ChlE | Tetrapyrrole, chlorophyll biosynthesis (aerobic) | *Synechococcus* sp. RS9917 | 92 | 100 |
| Oxygen dependent oxidase hemF |  | *Synechococcus* sp. MIT S9509 | 84 | 100 |
| radical SAM family variant BchE | Tetrapyrrole, chlorophyll biosynthesis (anaerobic) | *Synechococcus* sp. RS9916 | 92 | 97 |
| oxygen-independent oxidase hemN |  | *Synechococcus* sp. RS9917 | 77 | 98 |

| 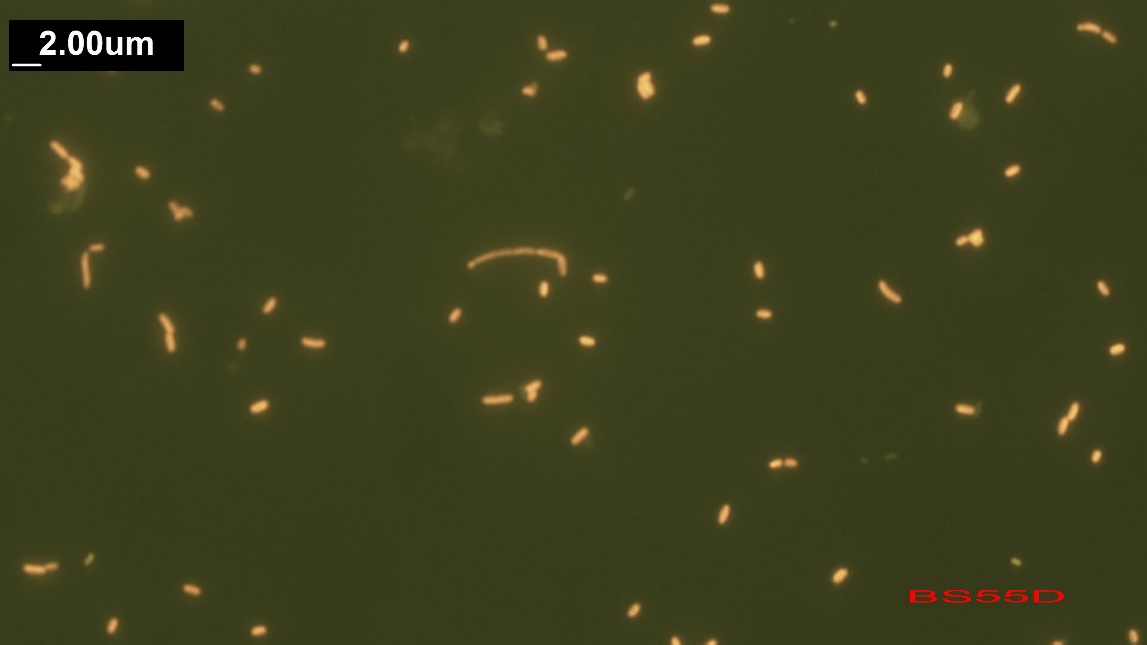 |
| --- |
|  |
| 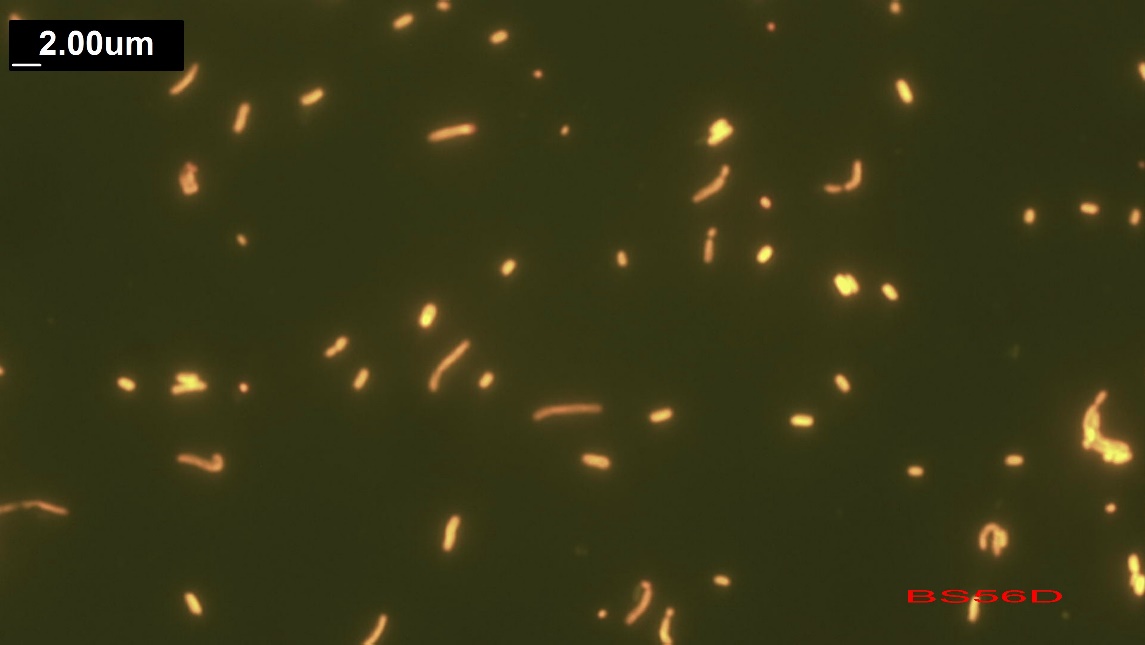 |

Fig. S1. *Synechococcus* strain BS55D (size: 1.30x0.69 µm) and *Synechococcus* BS56D (size: 1.69x0.79µm) isolated from the western gyre of Black Sea at 750 m. The elongated forms are 3-4 µm long. Longer filaments are visible in old cultures under light/oxic conditions (size: 16.6 x 0.36 µm). In the experiment the majority of the cells were short rods.

| 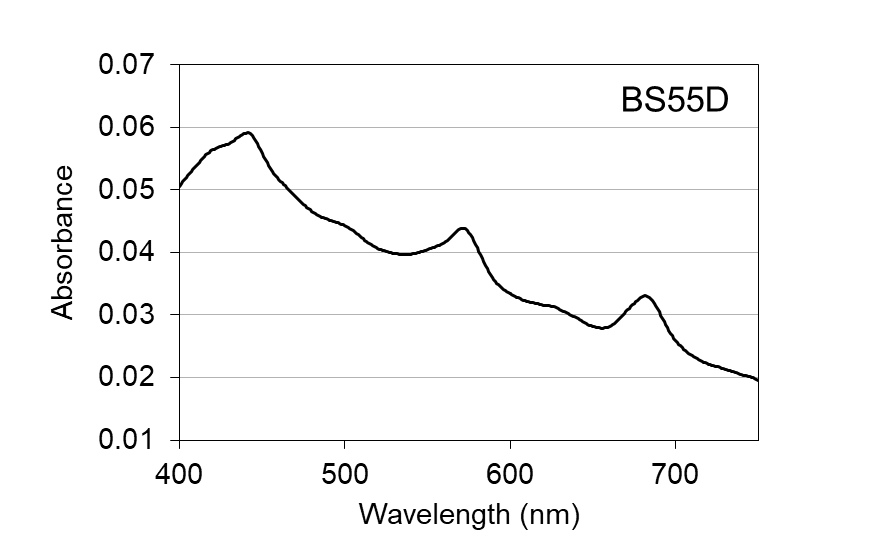 |
| --- |
| 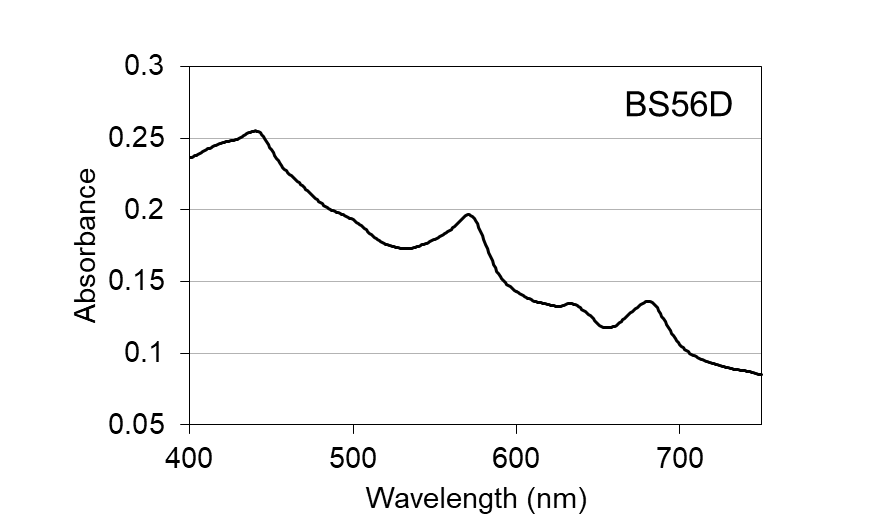 |

Fig. S2. Absorption spectra of the two *Synechococcus* strains BS55D (upper) and BS56D (lower). In vivo absorbance spectra of the diluted cultures were measured with a double monochromator spectrophotometer (SAFAS UVMC2) in the wavelength range 400 – 750 nm. Spectra were recorded with a 1 nm interval and 5 nm slit width in a quartz cuvette of 1 cm. 50µL of NaClO 10% was directly added in the cuvette and the samples read again to record the scatter. Absorbance due to scatter was subtracted from the spectra values at each wavelength for each sample.


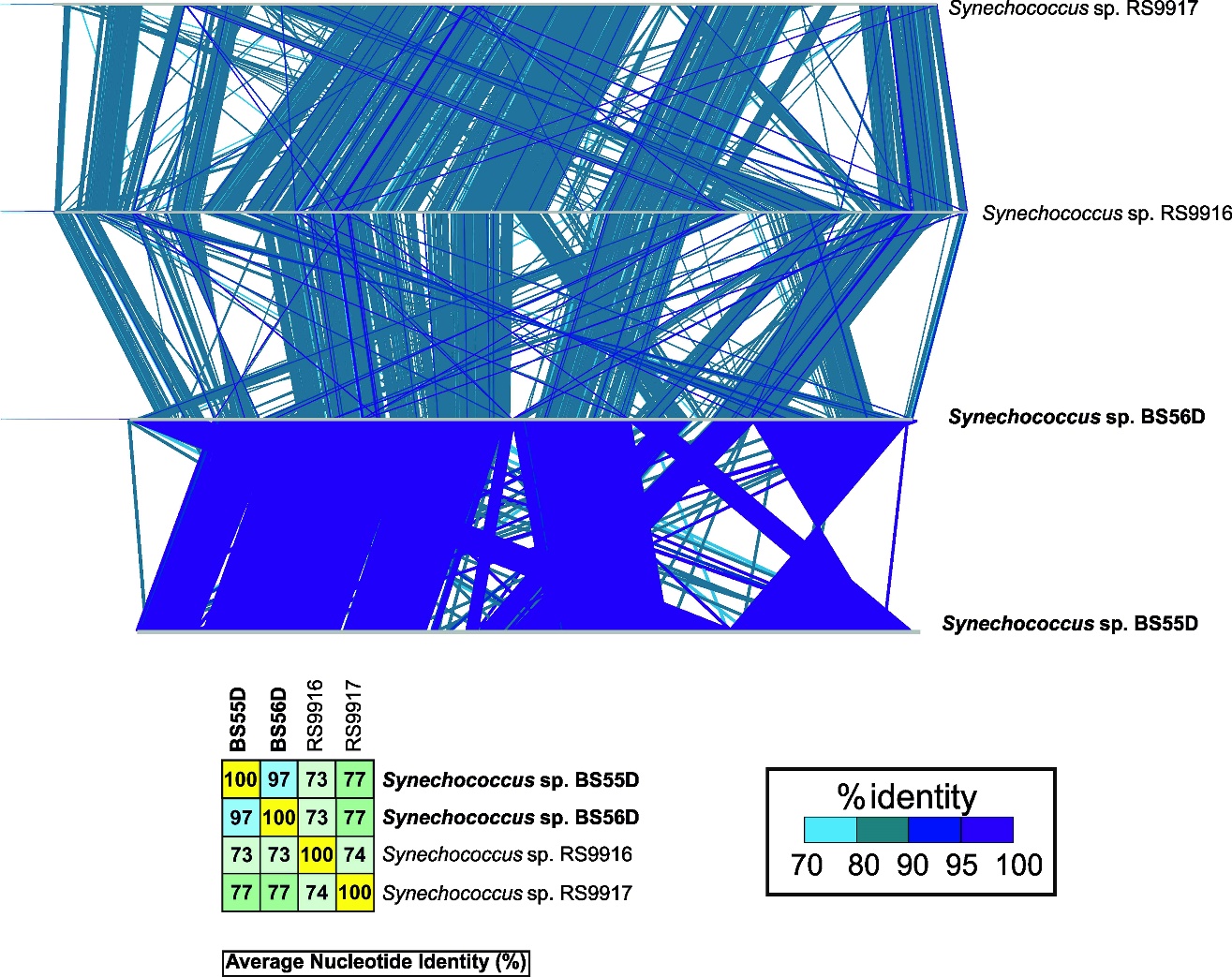


Fig. S3. Synteny plots between Red Sea strains *Synechococcus* sp. RS9917/RS9916 and Black Sea strain *Synechococcus* sp. BS56D. Comparison made with BLASTN with >70% identity hits and >100 bp of alignment lengths. Average Nucleotide Identity (ANI) between Red Sea and Black Sea strains is also shown.

The comparison of the synteny between Red Sea strains vs Black Sea deep strains revealed a very similar genetic organization between RS9917/RS9916 and BS55D/BS56D strains, even though ANI (Average Nucleotide Identity) between Black Sea and Red Sea strains was lower than 80%. This illustrate the adaptability of these organisms as it is encoded in the genome through standing ancestral pathways.
